# Microbiome-Based Therapies in Ulcerative Colitis: Mechanisms, Clinical Evidence, and a Precision-Medicine Framework

**DOI:** 10.3390/biomedicines14050969

**Published:** 2026-04-23

**Authors:** Philippe Pinton

**Affiliations:** Ferring Pharmaceuticals, Amager Strandvej 405, 2770 Kastrup, Denmark; philippe.pinton@shiroito.co.jp

**Keywords:** ulcerative colitis management, microbial therapeutics, fecal microbiota transplantation, defined consortia, gut ecology, immune modulation, metabolic pathways, precision medicine

## Abstract

Microbiome-based therapies are reshaping the therapeutic landscape for ulcerative colitis (UC), offering new avenues for disease management beyond conventional immunomodulatory and biologic treatments. UC remains a chronic, relapsing condition with significant unmet clinical needs, as many patients fail to achieve sustained remission or experience adverse effects with current therapies. The gut microbiome has emerged as a central contributor to UC pathogenesis, influencing epithelial barrier integrity, immune homeostasis, and metabolic signaling. Interventions such as fecal microbiota transplantation (FMT) and defined microbial consortia have demonstrated proof-of-concept efficacy in early-phase clinical trials, each leveraging distinct mechanistic strategies. FMT, as a broad ecological intervention, restores microbial diversity and functional redundancy, potentially addressing multiple pathogenic mechanisms simultaneously. In contrast, defined consortia enable precise targeting of specific metabolic and immunological pathways, including short-chain fatty acid production, bile-acid remodeling, epithelial barrier reinforcement, immune modulation, and succinate degradation. Recent clinical evidence suggests that consortia with broader mechanistic coverage may achieve more consistent biological activity than narrowly focused designs. This review synthesizes mechanistic and clinical insights across broad and defined microbial consortia, integrates evidence from randomized controlled trials and early-phase LBP studies, and outlines a precision-medicine framework to guide therapy selection. We highlight the importance of aligning therapeutic mechanisms with patient-specific microbial, metabolic, and immune profiles, and discuss future directions including biomarker-guided stratification, hybrid consortia, and adaptive trial designs. Advancing both broad and defined approaches, while incorporating ecological principles, mechanistic understanding, and patient stratification, will be essential to realizing the full therapeutic potential of microbiome-based therapies in UC.

## 1. Introduction

Ulcerative colitis (UC) is a chronic, immune-mediated inflammatory disease of the colon characterized by relapsing–remitting mucosal inflammation, progressive cumulative burden, and substantial impact on quality of life. Its global prevalence continues to rise, particularly in industrialized regions, with increasing incidence in newly industrializing countries. The disease course is heterogeneous, ranging from mild intermittent symptoms to severe, refractory inflammation requiring hospitalization or colectomy, and is associated with significant psychosocial and functional impairment [1]. Recent epidemiological analyses indicate that UC incidence continues to rise globally, with particularly rapid increases in newly industrializing regions. Despite advances in biologics and small molecules, up to 30–40% of patients fail to achieve durable remission, and colectomy rates remain substantial in refractory disease. These trends underscore the need for therapeutic approaches that address upstream ecological and metabolic drivers of inflammation rather than solely targeting downstream immune pathways.

Therapeutic goals in UC have evolved toward achieving and maintaining clinical remission, endoscopic mucosal healing, and prevention of disease progression. Contemporary management strategies, as outlined in the ECCO 2022 [2] and ACG 2025 [3] guidelines, rely on a step-up or accelerated step-up approach incorporating 5-aminosalicylates, corticosteroids, immunomodulators, biologics, and targeted small molecules. Despite these advances, a substantial proportion of patients remain refractory, lose response over time, or experience treatment-limiting adverse events [4]. This persistent unmet need has catalyzed interest in therapeutic modalities that target upstream drivers of disease rather than downstream inflammatory pathways.

A growing body of evidence implicates gut microbial dysbiosis as a central contributor to UC pathogenesis, influencing epithelial barrier integrity [5,6], immune homeostasis [7,8], and metabolic signaling [9,10]. This has led to the development of microbiome-based therapies, which span a spectrum from broad microbial consortia to rationally designed, strain-defined products.

Fecal microbiota transplantation (FMT) represents the broadest approach, transferring a complex microbial ecosystem from a healthy donor to restore diversity and functional redundancy [11,12]. Randomized trials have demonstrated proof-of-concept efficacy in UC [13,14,15], but clinical deployment is constrained by variability in donor material, challenges in standardization, and safety considerations, including risks of pathogen transmission [16]. International consensus statements have emphasized the need for rigorous donor screening and standardized stool banking procedures [17,18].

In contrast, rationally designed live biotherapeutic products (LBPs) consist of single or multiple well-characterized bacterial strains produced from clonal cell banks. These defined consortia aim to target specific mechanistic pathways implicated in UC—such as short-chain fatty acid production [19], bile-acid metabolism [20,21], epithelial barrier reinforcement [6], immune modulation [22], or succinate-driven inflammation [23,24]—while offering improved consistency, scalability, and regulatory oversight. Recent analyses highlight the growing maturity of the LBP regulatory framework [25] and the emergence of next-generation consortia such as MH002 [26], SER-287 [27], SER-301 [28,29], VE202 [30], and rationally engineered synthetic communities [31].

Across this therapeutic landscape, precision medicine is emerging as a critical paradigm. Inter-individual variability in microbiome composition, immune signatures, metabolic profiles, and clinical phenotypes suggests that no single microbial intervention will be universally effective. Multi-omics studies have demonstrated distinct microbial and metabolic states associated with disease activity and treatment response [32,33,34]. Early evidence indicates that baseline microbial configurations and host immune markers may predict response to both FMT and defined consortia [35,36], underscoring the need for stratification strategies to guide therapy selection. Integrating microbiome profiling, immune and metabolic biomarkers, and clinical characteristics into therapeutic decision-making may enable more targeted and effective deployment of microbiome-based therapies [37,38].

Previous reviews have summarized the development of microbiome-targeted interventions in inflammatory bowel disease [39,40,41]. However, there remains a need for a focused perspective that synthesizes mechanistic insights, clinical trial evidence, and strategic considerations specific to UC. The evidence synthesized in this review includes randomized controlled trials of FMT, early-phase studies of defined microbial consortia, and multi-omics analyses linking microbial, metabolic, and immune signatures to therapeutic response. The aim of this review is therefore to (i) provide an updated overview of microbiome-based therapies in clinical development, spanning broad and defined consortia; (ii) map these therapies to the mechanistic pathways they target; (iii) extract strategic insights from recent clinical trials to inform future therapeutic design; and (iv) outline the role of precision medicine and patient stratification in advancing the field toward clinically actionable, mechanism-driven interventions.

## 2. Literature Search Strategy

This review was developed using a structured literature search designed to capture mechanistic, translational, and clinical evidence related to microbiome-based therapies in ulcerative colitis. Searches were conducted in PubMed, Embase, Web of Science, and ClinicalTrials.gov, covering publications from inception to March 2026. Search terms included combinations of “ulcerative colitis”, “microbiome”, “dysbiosis”, “fecal microbiota transplantation”, “live biotherapeutic product”, “defined consortium”, “synthetic community”, “microbial metabolite”, “succinate”, “short-chain fatty acids”, “bile acids”, “engraftment”, and “precision medicine”.

Randomized controlled trials of FMT, early-phase studies of defined microbial consortia, mechanistic investigations of microbial pathways implicated in UC, and multi-omics analyses linking microbial or metabolic signatures to treatment response were included. Non-English articles, conference abstracts without full data, and studies lacking relevance to UC were excluded.

Regulatory documents, consensus statements, and industry-sponsored trial updates were reviewed to contextualize the evolving landscape of LBP development. Reference lists of key articles were manually screened to identify additional relevant studies. The objective of this search strategy was to ensure comprehensive coverage of mechanistic pathways, therapeutic modalities, and precision-medicine frameworks relevant to microbiome-based interventions in UC.

## 3. Microbiome-Based Therapies and the Mechanistic Pathways They Target

Microbiome-based therapies for UC act through multiple mechanistic pathways that converge on epithelial barrier integrity, immune regulation, metabolic signaling, and ecological restoration. Although broad microbial consortia and strain-defined LBPs differ in composition and design philosophy, both aim to modulate upstream biological processes that contribute to chronic mucosal inflammation.

### 3.1. Epithelial Barrier Integrity

Disruption of the intestinal epithelial barrier is a hallmark of UC, characterized by impaired tight-junction architecture, increased permeability, and reduced mucus layer integrity. Several microbial taxa depleted in UC, including *Faecalibacterium prausnitzii*, *Akkermansia muciniphila*, and butyrate-producing *Clostridia*, play key roles in maintaining epithelial homeostasis through the production of short-chain fatty acids (SCFAs), regulation of tight-junction proteins, and stimulation of mucin synthesis [5,6]. Microbiome-based therapies aim to restore these functions by reintroducing SCFA-producing organisms or by promoting ecological conditions that support their expansion.

### 3.2. Immune Modulation

Gut microbes influence both innate and adaptive immune responses through microbial metabolites, cell-surface structures, and interactions with pattern-recognition receptors. Dysbiosis in UC is associated with reduced regulatory T-cell (Treg) activity, increased Th17-driven inflammation, and heightened responsiveness to microbial antigens [7,8]. Microbial consortia designed to enhance Treg induction, modulate dendritic-cell activation, or suppress pro-inflammatory cytokine production aim to rebalance these immune pathways. Specific strains have been shown to promote IL-10 production, enhance Treg differentiation, or attenuate NF-κB activation, providing mechanistic rationale for their inclusion in defined LBPs [22,42].

### 3.3. Metabolic Pathways

Metabolic dysfunction is increasingly recognized as a driver of mucosal inflammation in UC. Key pathways include:

- SCFA production, particularly butyrate, which supports epithelial energy metabolism, reinforces barrier function, and exerts anti-inflammatory effects [9,10,19].
- Bile-acid metabolism, where reduced microbial conversion of primary to secondary bile acids contributes to impaired FXR and TGR5 signaling, promoting inflammation [20,21].
- Succinate accumulation, a pro-inflammatory metabolite elevated in UC that activates SUCNR1 signaling and amplifies mucosal immune activation [23,24,25,43].

Microbiome-based therapies aim to restore these metabolic networks by reintroducing key metabolic taxa or by reconstructing functional pathways through defined consortia.

### 3.4. Ecological Restoration and Functional Redundancy

UC is characterized by reduced microbial diversity, loss of keystone taxa, and diminished functional redundancy. FMT seeks to restore ecological stability by transferring a complete microbial ecosystem capable of re-establishing trophic networks, competitive exclusion, and metabolic complementarity [11,12,13,14,15]. Defined consortia, while more targeted, aim to recreate essential ecological functions using curated combinations of strains with complementary metabolic and immunomodulatory properties. The degree to which ecological restoration is required for clinical benefit remains an area of active investigation [44].

### 3.5. Engraftment Dynamics

Successful microbial engraftment is a prerequisite for sustained therapeutic activity. Engraftment depends on host factors (diet, inflammation, immune tone), ecological context (niche availability, competition), and microbial traits (fitness, metabolic compatibility). FMT exhibits broad engraftment patterns but with substantial inter-individual variability [13,14,15]. Defined LBPs demonstrate more predictable engraftment trajectories, though long-term persistence varies across products [26,27,28,29,30,31]. Understanding engraftment determinants is essential for optimizing dosing strategies, pre-conditioning regimens, and patient selection.

### 3.6. Mechanistic Divergence Between Broad and Defined Consortia

Broad microbial consortia such as FMT exert multi-pathway effects through ecological restructuring, restoration of metabolic networks, and modulation of immune responses. In contrast, defined LBPs target specific pathways with greater precision, enabling mechanistic attribution and regulatory standardization. These differences influence therapeutic consistency, safety profiles, and the potential for precision-medicine applications. The relative advantages of each approach likely depend on patient-specific microbial and immunological contexts.

These mechanistic distinctions between broad and defined consortia are illustrated in the following figures.

**Figure 1:**
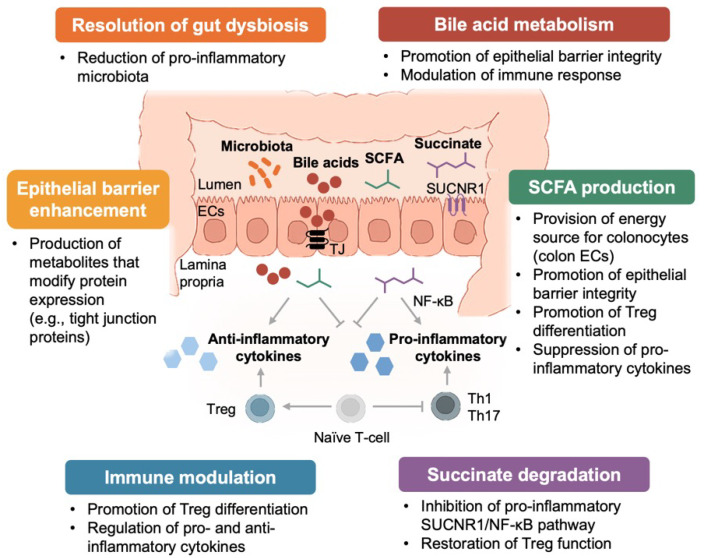
Mechanistic pathways through which microbiome-based therapies influence inflammation and mucosal homeostasis in ulcerative colitis. This figure summarizes the major functional axes modulated by microbial interventions. Short-chain fatty acid (SCFA)-producing taxa support epithelial barrier integrity, provide energy to colonocytes, promote regulatory T-cell differentiation, and suppress pro-inflammatory cytokines. Bile-acid-modifying microbes restore secondary bile acids and normalize FXR/TGR5 signaling, contributing to epithelial protection and immune regulation. Succinate-degrading strains reduce pro-inflammatory SUCNR1/NF-κB signaling and help restore Treg function. Additional mechanisms include reinforcement of tight-junction structure, modulation of mucosal immune tone, and reduction in pro-inflammatory microbial communities. Together, these pathways illustrate how broad consortia (e.g., FMT) can restore multiple functions simultaneously, whereas defined consortia typically target specific mechanistic deficits.

**Figure 2:**
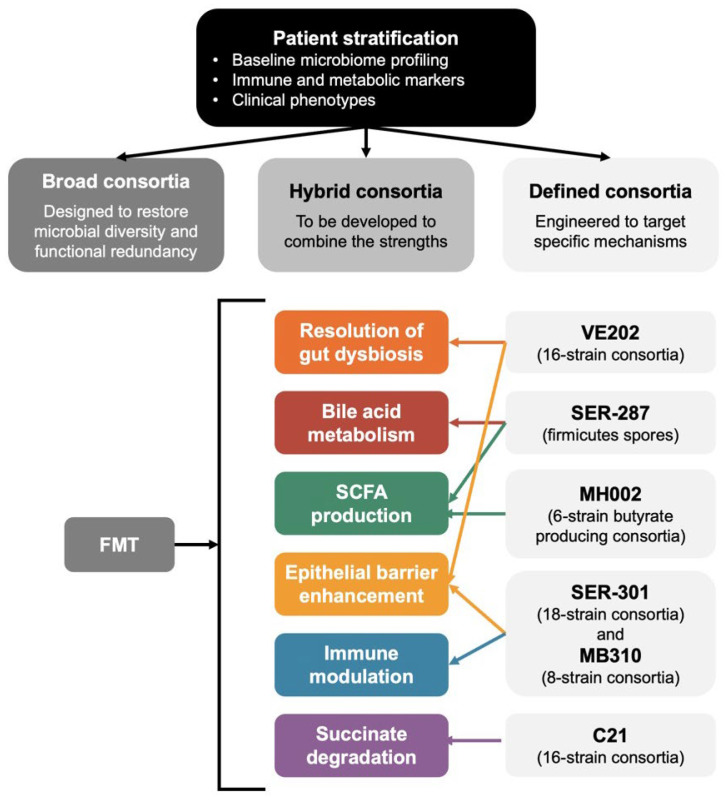
Therapeutic categories of microbiome-based interventions and their alignment with patient stratification frameworks. This figure illustrates how baseline microbiome profiling, immune and metabolic markers, and clinical phenotypes can guide the selection of broad, hybrid, or defined microbial consortia. Broad consortia (e.g., FMT) aim to restore ecological diversity and functional redundancy. Defined consortia deliver targeted mechanistic functions such as SCFA production (MH002), bile-acid remodeling (SER-287), epithelial-barrier enhancement (SER-301, MB310), immune modulation (C21), or succinate degradation. Hybrid consortia represent emerging designs that combine rationally selected strains with broader ecological functions to integrate precision with resilience. The figure highlights how mechanistic alignment between patient phenotype and microbial function can support precision-medicine approaches in ulcerative colitis.

Figure 1 and Figure 2 summarize the mechanistic pathways targeted by microbiome-based therapies and the two major therapeutic categories, respectively.

## 4. Clinical Evidence and Strategic Insights from Microbiome-Based Therapies

Clinical evaluation of microbiome-based therapies in UC has progressed along two parallel trajectories: randomized controlled trials of fecal microbiota transplantation (FMT) and early-phase studies of defined microbial consortia. Together, these studies provide proof-of-concept evidence that targeted modulation of the gut microbiome can induce clinical and endoscopic improvements in selected patient populations.

### 4.1. Evidence from Fecal Microbiota Transplantation

Multiple randomized controlled trials have demonstrated that FMT can induce clinical remission and endoscopic improvement in UC, although outcomes vary across studies due to differences in donor selection, delivery route, dosing frequency, and patient characteristics. Trials using intensive multi-donor regimens have reported higher remission rates, suggesting that ecological breadth and sustained microbial pressure may be important determinants of therapeutic efficacy. Donor-dependent effects have been consistently observed, with specific microbial signatures, including enrichment of butyrate-producing taxa and increased community diversity, associated with clinical response [45]. Despite these encouraging findings, heterogeneity in trial design and variability in donor material limit the generalizability of results, and safety considerations continue to constrain widespread clinical adoption.

### 4.2. Evidence from Defined Microbial Consortia

Defined microbial consortia have been evaluated in several early-phase clinical studies, each designed to target specific mechanistic pathways implicated in UC. These products vary in composition, dosing strategy, and mechanistic intent, ranging from SCFA-producing consortia to strains engineered to modulate immune or metabolic pathways.

Products such as SER-287, SER-301 and VE202 illustrate this diversity in mechanistic intent and clinical performance.

MH002, a six-strain rationally designed consortium, has demonstrated safety and signals of efficacy in a Phase 2a randomized controlled study, with consistent engraftment and improvements in clinical, endoscopic, and biomarker outcomes [26,46].

Phase 1 and 2 studies have demonstrated that defined consortia are generally well tolerated, with predictable pharmacokinetic and engraftment profiles. Some products have shown signals of clinical activity, including reductions in inflammatory biomarkers, improvements in endoscopic scores, and shifts toward microbial communities associated with health. However, efficacy outcomes have been variable across products, reflecting differences in mechanistic breadth, strain selection, and ecological compatibility with the host microbiome.

### 4.3. Engraftment and Biological Activity

Across both FMT and defined consortia, microbial engraftment has emerged as a key determinant of therapeutic effect. Responders typically exhibit higher levels of donor-derived or consortium-derived taxa, along with restoration of metabolic pathways such as butyrate production, secondary bile-acid synthesis, and succinate degradation. Non-responders often display ecological resistance, characterized by persistent dysbiosis, inflammatory metabolic signatures, or failure of introduced strains to establish within the gut ecosystem.

### 4.4. Safety Considerations

Safety profiles differ between broad and defined microbial interventions. FMT carries risks related to pathogen transmission, donor variability, and challenges in standardization, necessitating rigorous screening and controlled manufacturing processes. Defined consortia offer improved reproducibility and regulatory oversight but raise questions regarding long-term engraftment, ecological interactions, and theoretical risks such as horizontal gene transfer. To date, clinical trials of defined LBPs have reported favorable safety profiles, with adverse events generally mild and transient.

### 4.5. Interpretation of Clinical Evidence

Taken together, clinical studies demonstrate that microbiome-based therapies can induce meaningful biological and clinical effects in UC, but outcomes depend on multiple factors, including donor or strain selection, mechanistic breadth, dosing strategy, and host ecological context. FMT provides strong proof-of-concept for ecological restoration, while defined consortia offer mechanistic precision and regulatory scalability. The variability in clinical outcomes underscores the need for improved patient stratification, optimized dosing regimens, and deeper understanding of engraftment biology to enhance therapeutic consistency.

Representative examples of these defined microbial consortia and synthetic communities are summarized in Table 1. Table 1 summarizes the main clinical characteristics of microbiome-based therapies currently evaluated in ulcerative colitis, including their composition, mechanisms of action, trial design, patient populations, treatment regimens, preconditioning strategies, engraftment profiles, and key outcomes.

## 5. Therapeutic Positioning, Clinical Scenarios, and Decision Frameworks

Microbiome-based therapies for UC can be positioned along a continuum defined by mechanistic breadth, ecological impact, regulatory complexity, and potential for personalization. Broad microbial consortia such as FMT exert wide-ranging effects across multiple biological pathways, whereas defined LBPs offer targeted modulation of specific mechanisms with greater standardization and reproducibility. Understanding the strengths and limitations of each approach is essential for aligning therapeutic strategies with patient-specific needs and disease biology.

### 5.1. Broad Ecological Interventions

FMT represents the most comprehensive ecological intervention, capable of restoring microbial diversity, re-establishing functional redundancy, and modulating multiple metabolic and immune pathways simultaneously. This breadth may be advantageous in patients with profound dysbiosis, extensive metabolic disruption, or inflammatory phenotypes driven by multiple upstream defects. However, variability in donor material, challenges in standardization, and safety considerations limit its scalability and regulatory acceptance. The heterogeneity of clinical responses observed across trials underscores the need for improved donor selection, optimized dosing strategies, and patient stratification.

### 5.2. Mechanistically Targeted Defined Consortia

Defined LBPs offer a complementary strategy focused on precise modulation of specific pathways implicated in UC pathogenesis. These products are designed to deliver reproducible biological activity, predictable engraftment patterns, and controlled manufacturing processes. Mechanistic specificity may be particularly valuable in patients with identifiable metabolic or immunological defects, such as reduced SCFA production, impaired bile-acid conversion, or succinate-driven inflammation, where targeted interventions can restore discrete functional pathways. However, narrower mechanistic scope may limit efficacy in patients with complex or multifactorial dysbiosis.

Recent analyses highlight the growing maturity of the LBP regulatory framework [25,49], supporting the development of standardized, reproducible microbial therapeutics.

### 5.3. Hybrid and Next-Generation Approaches

Emerging strategies aim to combine the ecological breadth of FMT with the precision and reproducibility of defined LBPs. These include rationally assembled multi-strain consortia designed to recapitulate key ecological functions, engineered communities with enhanced metabolic capabilities, and modular platforms that allow tailoring of strain composition to patient-specific biological profiles. Such hybrid approaches may offer a balance between mechanistic breadth and regulatory feasibility, though clinical validation remains ongoing.

### 5.4. Matching Therapeutic Modality to Patient Profile

Therapeutic positioning increasingly depends on aligning the mechanism of action with patient-specific microbial, metabolic, and immune signatures. Patients with severe dysbiosis, low microbial diversity, or broad metabolic disruption may benefit from ecological restoration strategies such as FMT or multi-strain consortia. Conversely, patients with preserved diversity but discrete functional deficits, such as impaired butyrate production, altered bile-acid metabolism, or elevated succinate, may be more responsive to targeted LBPs. Integrating baseline microbiome profiling, inflammatory markers, and metabolic signatures into clinical decision-making may enhance therapeutic precision and improve outcomes.

### 5.5. Strategic Considerations for Clinical Development

From a development perspective, broad consortia offer strong proof-of-concept for microbiome modulation but face challenges in standardization, regulatory approval, and scalability. Defined LBPs provide clearer mechanistic attribution and manufacturing control but must demonstrate sufficient breadth of activity to achieve consistent clinical benefit. Future progress will likely depend on optimizing strain selection, improving engraftment strategies, and incorporating biomarker-guided patient selection into trial design.

## 6. Precision Medicine and Patient Stratification in Microbiome-Based Therapies

Precision medicine is increasingly recognized as a critical framework for optimizing the use of microbiome-based therapies in UC. Inter-individual variability in microbial composition, metabolic activity, immune signatures, and clinical phenotype suggests that no single microbial intervention will be universally effective. Instead, therapeutic success is likely to depend on aligning the mechanism of action with patient-specific biological drivers.

### 6.1. Microbial Stratification

Baseline microbiome composition has emerged as a key determinant of response to both FMT and defined microbial consortia. Responders typically exhibit microbial configurations characterized by higher diversity, greater niche availability, or the presence of taxa compatible with engraftment of donor- or consortium-derived strains. In contrast, non-responders often display ecological resistance, persistent dysbiosis, or metabolic signatures associated with inflammation. Multi-omics studies have identified microbial and metabolic states associated with disease activity and treatment response [32,33,34,50], supporting the use of microbiome profiling to guide therapeutic selection.

### 6.2. Immune and Metabolic Biomarkers

Host immune tone and metabolic environment influence both engraftment and therapeutic activity. Elevated inflammatory cytokines, impaired Treg function, and metabolic disturbances such as reduced SCFA production or increased succinate levels may shape responsiveness to specific microbial interventions [33]. Early evidence indicates that baseline immune markers and metabolic signatures can predict response to FMT and defined consortia [32,34,35,36,51]. Integrating these biomarkers into clinical decision-making may enable more targeted deployment of microbiome-based therapies.

### 6.3. Integrating Clinical Phenotypes

Clinical characteristics, including disease extent, severity, treatment history, and comorbidities, also influence therapeutic outcomes. Patients with extensive dysbiosis, broad metabolic disruption, or refractory inflammation may benefit from ecological restoration strategies such as FMT or multi-strain consortia. Conversely, patients with preserved diversity but discrete functional deficits may be more responsive to targeted LBPs. Incorporating clinical phenotypes alongside microbial and immune biomarkers can refine patient stratification and improve therapeutic precision.

### 6.4. Toward Mechanism-Driven Therapeutic Selection

The convergence of microbial, immune, metabolic, and clinical data supports a mechanism-driven approach to therapy selection. Rather than applying microbiome-based therapies uniformly, precision medicine aims to match the therapeutic modality, broad ecological intervention, targeted consortium, or hybrid approach to the dominant biological drivers in each patient. This strategy has the potential to enhance efficacy, reduce variability in clinical outcomes, and accelerate the development of next-generation microbial therapeutics.

### 6.5. Implications for Clinical Trial Design

Precision-medicine principles are increasingly being incorporated into trial design for microbiome-based therapies. Baseline stratification using microbial or metabolic biomarkers, adaptive dosing strategies, and enrichment of biologically defined subgroups may improve the detection of therapeutic signals and clarify mechanisms of action. As the field evolves, integrating multi-omics profiling and mechanistic biomarkers into early-phase studies will be essential for identifying responders, optimizing strain selection, and guiding the development of more personalized interventions.

## 7. Future Directions

The field of microbiome-based therapeutics for UC is entering a phase of rapid maturation, driven by advances in microbial ecology, synthetic biology, multi-omics profiling, and regulatory science. Several key directions are likely to shape the next generation of interventions and accelerate their translation into clinical practice.

### 7.1. Rational Design of Next-Generation Consortia

Future microbial therapeutics will increasingly rely on rationally assembled consortia that combine ecological robustness with mechanistic specificity, supported by advances in metabolic modeling and synthetic ecology [52]. These products aim to recapitulate essential functions of a healthy microbiome, such as SCFA production, bile-acid conversion, and succinate degradation, while maintaining the reproducibility and safety required for pharmaceutical development. Advances in strain selection, metabolic modeling, and functional genomics will enable more precise engineering of microbial communities tailored to UC-specific biological defects.

### 7.2. Engineered and Synthetic Microbial Communities

Synthetic biology offers opportunities to design strains with enhanced metabolic capabilities, improved engraftment potential, or programmable immunomodulatory functions [53]. Engineered microbes may be used to deliver therapeutic metabolites, degrade pro-inflammatory molecules, or modulate host signaling pathways with high specificity, and modular synthetic communities could allow customization of strain composition based on patient-specific microbial or metabolic profiles [54]. Modular synthetic communities could allow customization of strain composition based on patient-specific microbial or metabolic profiles, supporting a more personalized therapeutic approach.

### 7.3. Enhancing Engraftment and Ecological Stability

Engraftment remains a central challenge for both FMT and defined consortia. Future strategies may include dietary pre-conditioning, targeted niche creation, co-administration of metabolic substrates, or temporal sequencing of strains to promote ecological compatibility. Understanding host–microbe and microbe–microbe interactions will be essential for optimizing engraftment durability and ensuring sustained therapeutic benefit.

### 7.4. Biomarker-Guided Personalization

Precision-medicine frameworks will increasingly guide therapeutic selection, dosing, and monitoring. Multi-omics profiling, including metagenomics, metabolomics, transcriptomics, and immune phenotyping, may identify microbial or metabolic signatures predictive of response. Integrating these biomarkers into clinical workflows could enable real-time stratification, adaptive trial designs, and personalized therapeutic regimens that match microbial interventions to patient-specific biological drivers.

### 7.5. Regulatory and Manufacturing Innovation

As the regulatory landscape for LBPs continues to evolve, new frameworks will support the development of more complex microbial products, including multi-strain consortia and engineered communities, and harmonization of international standards will be essential [49]. Advances in manufacturing, quality control, and potency assays will be critical for ensuring consistency, safety, and scalability. Harmonization of international regulatory standards may further accelerate global development and clinical adoption.

### 7.6. Combination Therapies and Integrated Care Models

Microbiome-based therapies may ultimately be integrated with existing UC treatments, including biologics, small molecules, and dietary interventions. Combination strategies could enhance efficacy by simultaneously targeting immune pathways and upstream ecological or metabolic dysfunction. Understanding how microbial therapeutics interact with immunomodulators, anti-inflammatories, and nutritional interventions will be essential for designing synergistic treatment regimens.

### 7.7. Long-Term Outcomes and Real-World Evidence

Long-term follow-up studies and real-world evidence will be needed to assess durability of response, ecological stability, safety, and impact on disease progression. Registries, post-marketing surveillance, and longitudinal cohort studies will provide critical insights into long-term engraftment, metabolic reprogramming, and sustained clinical benefit. These data will inform best practices for patient selection, monitoring, and therapeutic optimization.

## 8. Conclusions

Microbiome-based therapies represent a rapidly evolving therapeutic class with the potential to address upstream ecological and metabolic drivers of ulcerative colitis. Evidence from randomized trials of FMT and early-phase studies of defined microbial consortia demonstrates that targeted modulation of the gut microbiome can induce meaningful biological and clinical effects in selected patients. However, variability in therapeutic response underscores the complexity of host–microbe interactions and the need for more refined approaches to patient selection, dosing, and mechanistic targeting.

Broad ecological interventions such as FMT offer multi-pathway activity and strong proof-of-concept for microbiome restoration but face challenges related to standardization, donor variability, and safety. Defined LBPs provide mechanistic precision, reproducibility, and regulatory scalability yet may require careful alignment with patient-specific microbial and metabolic contexts to achieve consistent efficacy. Emerging hybrid and engineered microbial communities may help bridge the gap between ecological breadth and mechanistic specificity.

Precision-medicine frameworks integrating microbial, metabolic, immune, and clinical biomarkers will be essential for guiding therapeutic selection and optimizing outcomes. Advances in multi-omics profiling, mechanistic modeling, and biomarker-guided trial design are expected to accelerate the development of next-generation microbial therapeutics and support more personalized interventions. As the field matures, long-term follow-up studies, real-world evidence, and harmonized regulatory standards will be critical for ensuring safety, durability, and clinical integration.

Microbiome-based therapies are poised to become an important component of the therapeutic landscape in UC. Continued progress will depend on mechanistic insight, rigorous clinical evaluation, and the development of precision-medicine strategies that match microbial interventions to the biological drivers of disease in each patient.

## Figures and Tables

**Table 1:** Clinical characteristics of microbiome-based therapies evaluated in UC. The table details the composition, mechanistic rationale, clinical development stage, patient populations, treatment regimens, preconditioning approaches, engraftment and microbiome outcomes, and key findings of representative microbiome-based interventions, including MH002, SER 287, SER 301, VE202, and emerging synthetic microbial communities.

Therapy	Type/Consortium	MOA	Phase	N; f/u	Severity	Dose; Duration	Precond.	Engraft./Microbiome	Key Findings	ID/Refs
**MH002**	Rationally designed 6-strain live biotherapeutic consortium	Immune modulation Epithelial repair Metabolic restoration (↑ SCFA; ↓ inflammatory cytokines)	Phase 2a (randomized, double-blind, placebo-controlled)	N = 45; 8w induction + 8w extension	Mild–mod. UC	400 mg QD × 8w	None beyond standard of care	Stable engraftment of all six strains Restoration of microbial diversity	Primary endpoint: safety met Efficacy signals: clinical remission, endoscopic improvement, biomarkers	EudraCT 2020-004355-33 [26,46,47]
**SER-287**	Spore-based consortium derived from healthy donor microbiota	Ecological restoration via spore-forming commensals ↑ butyrate Treg induction	Phase 2b (randomized, double-blind, placebo-controlled)	N = 203; f/u NR	Mild–mod. UC	Oral capsules; induction → maintenance	Vancomycin in selected arms	Engraftment improved with antibiotic preconditioning Transient microbiome shifts	Phase 2b: did not meet primary endpoint Earlier efficacy signals in subgroups	NCT03759041 [48]
**SER-301**	Fully defined rationally designed consortium	SCFA restoration Bile-acid remodeling Anti-inflammatory metabolic modulation	Phase 1b (safety + efficacy)	N = 15; f/u NR	Mild–mod. UC	Oral capsules QD × 10w	Vancomycin	High engraftment of targeted strains Metabolic normalization	Safety demonstrated Partial clinical benefit; no full remissions	ACTRN12620000963921 [29]
**VE202**	Rationally designed multi-strain consortium	Defined commensals modulate mucosal T-cell responses ↑ Treg ↓ Th17	Phase 1/2	P1: N = 31; P2: N = 114; f/u NR	Mild–mod. UC	Oral capsules; several weeks	Vancomycin in some arms	Sustained colonization of core strains Immune signature normalization	Not superior to placebo on primary/secondary endpoints Safety confirmed	NCT05370885 [30]
**Synthetic consortia**	Rationally engineered multi-strain communities	SCFA production Succinate degradation Barrier support Immune modulation	Preclin.	Preclin.	Preclin.	Oral consortia; daily dosing	Often with antibiotic preconditioning	Stable colonization Restoration of metabolic balance	Comparable efficacy to FMT in preclinical models	[31]

Abbreviations: MOA, mode of action; f/u, follow-up; NR, not reported; UC, ulcerative colitis; SCFA, short-chain fatty acids; Treg, regulatory T cells; Th17, T helper 17 cells; FMT, fecal microbiota transplantation; QD, once daily; w, week(s); Preclin., preclinical.

## Data Availability

No new data were generated or analyzed in support of this Perspective.

## Abbreviations

The following abbreviations are used in this manuscript:

ACG	American College of Gastroenterology
ECCO	European Crohn’s and Colitis Organisation
FMT	Fecal microbiota transplantation
LBP	Live biotherapeutic product
SCFA	Short-chain fatty acid
UC	Ulcerative colitis
